# The Primary Enveloped Virion of Herpes Simplex Virus 1: Its Role in Nuclear Egress

**DOI:** 10.1128/mBio.00825-17

**Published:** 2017-06-13

**Authors:** William W. Newcomb, Juan Fontana, Dennis C. Winkler, Naiqian Cheng, J. Bernard Heymann, Alasdair C. Steven

**Affiliations:** a Laboratory of Structural Biology Research, National Institute of Arthritis and Musculoskeletal and Skin Diseases, National Institutes of Health, Bethesda, Maryland, USA; b Astbury Centre for Structural Molecular Biology and Faculty of Biological Sciences, University of Leeds, Leeds, United Kingdom; University of Maryland, College Park

**Keywords:** HSV capsid, cryo-electron microscopy, cryo-electron tomography, nuclear egress, nuclear egress complex, nuclear envelope, nuclear export

## Abstract

Many viruses migrate between different cellular compartments for successive stages of assembly. The HSV-1 capsid assembles in the nucleus and then transfers into the cytoplasm. First, the capsid buds through the inner nuclear membrane, becoming coated with nuclear egress complex (NEC) protein. This yields a primary enveloped virion (PEV) whose envelope fuses with the outer nuclear membrane, releasing the capsid into the cytoplasm. We investigated the associated molecular mechanisms by isolating PEVs from US3-null-infected cells and imaging them by cryo-electron microscopy and tomography. (pUS3 is a viral protein kinase in whose absence PEVs accumulate in the perinuclear space.) Unlike mature extracellular virions, PEVs have very few glycoprotein spikes. PEVs are ~20% smaller than mature virions, and the little space available between the capsid and the NEC layer suggests that most tegument proteins are acquired later in the egress pathway. Previous studies have proposed that NEC is organized as hexamers in honeycomb arrays in PEVs, but we find arrays of heptameric rings in extracts from US3-null-infected cells. In a PEV, NEC contacts the capsid predominantly via the pUL17/pUL25 complexes which are located close to the capsid vertices. Finally, the NEC layer dissociates from the capsid as it leaves the nucleus, possibly in response to pUS3-mediated phosphorylation. Overall, nuclear egress emerges as a process driven by a program of multiple weak interactions.

## IMPORTANCE

On its maturation pathway, the newly formed HSV-1 nucleocapsid must traverse the nuclear envelope, while respecting the integrity of that barrier. Nucleocapsids (125 nm in diameter) are too large to pass through the nuclear pore complexes that conduct most nucleocytoplasmic traffic. It is now widely accepted that the process involves envelopment/de-envelopment of a key intermediate—the primary enveloped virion. In wild-type infections, PEVs are short-lived, which has impeded study. Using a mutant that accumulates PEVs in the perinuclear space, we were able to isolate PEVs in sufficient quantity for structural analysis by cryo-electron microscopy and tomography. The findings not only elucidate the maturation pathway of an important human pathogen but also have implications for cellular processes that involve the trafficking of large macromolecular complexes.

## INTRODUCTION

Herpes simplex virus 1 (HSV-1) is the archetypal member of the *Herpesviridae*, an extensive family of viruses that infect hosts throughout the animal kingdom. Eight herpesviruses cause diseases in humans, including skin lesions, encephalitis, and cancers. Herpesviruses have large genomes of double-stranded DNA (152 kbp in the case of HSV-1) and need correspondingly large and elaborate virions to accommodate them. In the infected cell nucleus, HSV-1 replicates its genome and assembles its capsid, a 125-nm-diameter *T* = 16 icosahedral particle built from 10 different proteins (1). Subsequently, the nucleocapsid is transferred into the cytoplasm and goes on to acquire its tegument—a heterogeneous protein coating—and its envelope.

The HSV-1 capsid is too large to exit the nucleus via a nuclear pore, which has an exclusion limit of about 40 nm (2): instead, it employs a two-step process of envelopment/de-envelopment, based on interactions between the capsid, the inner nuclear membrane (INM), and the nuclear egress complex (NEC), a heterodimer of two virally encoded proteins: a membrane protein (pUL34) and one with affinity for the capsid (pUL31) (3). First, the nucleocapsid buds into the perinuclear space (PNS), producing a primary enveloped virion (PEV). Second, the PEV envelope fuses with the outer nuclear membrane (ONM), releasing the nucleocapsid into the cytoplasm. A cellular process for nucleocytoplasmic transport of large RNA complexes has recently been described (4, 5) that is mechanistically similar. The present study addressed the structural basis for the HSV-1 nuclear egress pathway and, in particular, the structure of the PEV.

Herpesvirus assembly begins with the formation of a metastable precursor called the procapsid that matures via a transformation of its structure and its affinity for other proteins (1). The surface shells of the three maturation products—A-capsids (empty), B-capsids (retaining scaffold protein), and C-capsids (with packaged genome)—have essentially the same structure except that C-capsids have, on average, 4-fold more of the heterodimeric pUL17/pUL25 complex [also known as the C-capsid-specific complex (CCSC) (6) or the capsid vertex-specific complex (CVSC) (7)]. This distinction correlates with the relative alacrity with which C-capsids embark upon nuclear egress (8). It has been proposed that DNA packaging induces a subtle conformational change in the C-capsid that enhances the affinity of the CCSC binding sites (6).

Recent papers have described the structure and certain other properties of the NEC. *In vitro* binding experiments (9) and cryo-electron tomography (cryo-ET) of cells overexpressing the NEC (10) have shown that the NEC is sufficient for membrane budding. Its structure has been solved by X-ray crystallography for the HSV-1 protein (11) and for the closely related pseudo-rabies virus (PrV) (11, 12) and cytomegalovirus (13). However, it has not been clear how the NEC interacts with the capsid. On one hand, an interaction between pUL31 and the CCSC was inferred, based on coimmunoprecipitation and biochemical analyses of wild-type and mutant capsids (14). On the other hand, it has been reported that pUL31 can interact with the capsid in the absence of pUL25 (15, 16). Moreover, earlier pulldown experiments suggested an interaction between pUL31 and major capsid protein pUL19 (VP5) (17).

The first sighting of a PEV by electron microscopy (EM) of thin sections of infected cells dates from 1969 (18). Since then, some progress has been made (19–21), albeit hampered by the fact that PEVs are transient particles, difficult to isolate in bulk. More recently, it has been discovered that certain mutants block the transfer of PEVs into the cytoplasm so that they accumulate in the PNS (22–25). In this study, we used a mutation of this kind and developed a method based on classical techniques of nuclei isolation, disruption, and differential centrifugation to obtain preparations enriched in PEVs. While the yields are not high, they have allowed detailed structural analysis by electron microscopy. In addition to PEVs, our isolates contained sacs, also called herniations or invaginations. These are artificial compartments created by the bulging of tracts of INM back into the nucleus, and they contain PEVs. These observations have allowed us to propose a model for how the NEC interacts with the capsid via the CCSC.

## RESULTS

### Isolation of PEVs.

Nuclei were extracted from cells infected with a US3-null mutant and disrupted by sonication; this material was then fractionated by differential sedimentation. The fractions with the highest content of PEVs were identified by Western blotting with an anti-pUL31 antibody and negative-staining EM (see Fig. S1 in the supplemental material). PEVs have a distinctive morphology and could be readily identified for structural analysis by EM and ET.

10.1128/mBio.00825-17.1 FIG S1  Preparation of PEV-containing isolates. (A) Sucrose gradients (20% to 50%) of A, B, and C-capsids (tube 1); clarified lysate of US3-null-infected cells (tube 2). P denotes the position where PEVs band; V, purified virus (tube 3). (B) EM of a negatively stained field containing PEVs. Scale bar = 100 nm. The star denotes a free capsid. (C) Western blotting of gradient fractions with anti-pUL31 antibodies. The P band in panel A2 was run on a second sucrose gradient, fractionated, subjected to Western blotting, and probed with anti-pUL31. Fraction 15 is the pUL31 control. The underlining bar marks PEV-containing fractions 6 and 7. We infer that the pUL31 detected in other fractions resides in INM fragments. Download FIG S1, TIF file, 2.2 MB. Copyright © 2017 Newcomb et al. 2017 Newcomb et al. This content is distributed under the terms of the Creative Commons Attribution 4.0 International license.

### Negative-staining EM.

**FIG 1:**
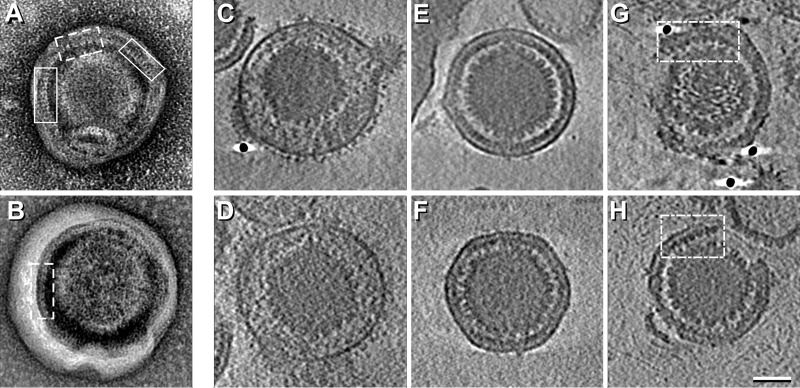
Electron micrographs and cryo-ET slices of PEVs. (A and B) Negatively stained micrographs showing “picket fence” motifs in the NEC layer. Tracts with the 6-nm repeat are boxed with continuous lines and tracts with the 10.5-nm repeat with dashed lines. (C to H) Examples of near-central slices through the respective density maps. (C and D) Mature virions. (E and F) Intact PEVs; (G and H) Broken PEVs. Tracts showing the 12-nm repeat are boxed. Scale bar = 50 nm.

Images of PEVs typically present three layers. The outermost layer is an envelope, derived from the inner nuclear membrane. The innermost layer is the capsid. Between them is an 8.5-nm-thick layer that we interpret as a monolayer of NEC molecules (Fig. 1). On many particles, a “picket fence” motif is visible in the NEC layer (examples are boxed in Fig. 1A and B). The repeat most commonly observed was at ~6 nm (5.9 ± 0.2 nm), and one was also detected at ~10.5 nm (10.3 ± 0.7 nm). These spacings correspond to two side-projections of a hexagonal array with a lattice constant of ~12 nm (Fig. S2A). Because picket fence tracts do not repeat at symmetry-related positions on opposite sides of a given particle, we infer that there is no global symmetry to the NEC lattice, which is, rather, a patchwork of locally ordered domains.

10.1128/mBio.00825-17.2 FIG S2  Regular p6 arrays and hexagonal close packing. (A) Schema explaining how 6-nm and 10.5-nm spacings can arise in honeycomb arrays with a lattice constant of 12 nm (the unit cell is delineated with black dashes). This is a regular p6 array in which the rings are hexamers of globular subunits. A reference point on each “subunit” is marked with a dot. (B) Hexagonal close packing of annular rings that are not necessarily hexamers and not necessarily in the same orientation. A reference point on each ring is marked with a dot. Both arrangements can have the appearance of honeycomb arrays when viewed at limited resolution or after averaging. Download FIG S2, TIF file, 0.5 MB. Copyright © 2017 Newcomb et al. 2017 Newcomb et al. This content is distributed under the terms of the Creative Commons Attribution 4.0 International license.

### Cryo-ET of PEVs.

**FIG 2:**
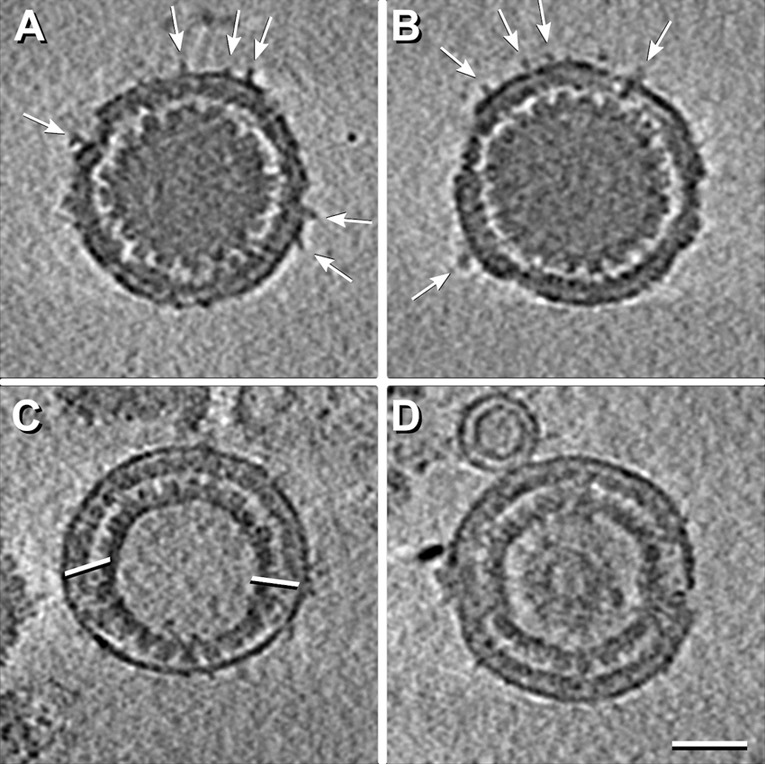
Central cryo-ET slices from reconstructions of four PEVs. The particles in panel A and B have a few protruding densities (white arrows). The PEV in panel C contains an A-capsid and has an unusually large fontanelle delimited by white bars, while the PEV in panel D contains a B-capsid. Scale bar = 50 nm.

Central slices through tomograms of two intact PEVs shown in Fig. 1E and F are compared with mature virions in Fig. 1C and D. A larger field is shown in Fig. S3. Mature virions are about 20% larger and more variable in size, i.e., 195 ± 11 nm (186 nm, in an earlier determination [26]) versus 158 ± 5 nm in diameter. Their envelopes are crowded with protruding glycoprotein spikes. In contrast, PEVs have very few spikes (Fig. 1E and F and 2A and B). Specifically, mature virions average 650 to 700 spikes per particle (26), whereas PEVs have only 16 ± 6 (range, 6 to 26).

10.1128/mBio.00825-17.3 FIG S3  A field of isolated PEVs imaged by cryo-ET. The field includes one mature virion (top right). Download FIG S3, TIF file, 2.5 MB. Copyright © 2017 Newcomb et al. 2017 Newcomb et al. This content is distributed under the terms of the Creative Commons Attribution 4.0 International license.

In mature virions, the space between the capsid and the envelope is occupied by the tegument, which is much thicker and more voluminous than the corresponding region of a PEV, which is largely filled by the NEC layer (cf. Fig. 1E and F and 1C and D).

Despite strenuous efforts to isolate intact PEVs, many (~55%) were missing part of their envelope/NEC layer complex (e.g., Fig. 1G and H), apparently reflecting breakage during isolation or incomplete budding. A picket fence motif was less commonly observed in tomographic slices than in negatively stained projections, suggesting that a slice has to be more precisely oriented than in the NEC array for the periodicity to be seen, but in some regions, the 12-nm repeat is evident (see, e.g., the boxed areas in Fig. 1G and H). Serial slices through the PEV tomogram in Fig. S4 show a NEC layer that is complete, apart from a gap ~40 nm across. We interpret this gap as a site where a PEV budded off and refer to it as a “fontanelle.”

10.1128/mBio.00825-17.4 FIG S4  Serial tomographic sections through a PEV. The slices are 1.6 nm apart. The fontanelle gap in the NEC layer is demarcated with arrows in panels 3 and 4 of the top row. Bar = 50 nm. We note that gaps were also observed in the NEC layers of at least some of the NEC-coated vesicles formed by *in vitro* invagination of giant unilamellar vesicles (see Fig. 4b of reference 9). Download FIG S4, TIF file, 2.8 MB. Copyright © 2017 Newcomb et al. 2017 Newcomb et al. This content is distributed under the terms of the Creative Commons Attribution 4.0 International license.

With tomograms, the kind of capsid present in a given PEV is readily identifiable. In our data set (*n* = 101), the majority (82%) contain C-capsids (e.g., Fig. 1E and F), but we also observed a few with A-capsids (~13%; e.g., Fig. 2C) or B-capsids (~5%; e.g., Fig. 2D).

### Cryo-EM reconstruction of PEVs.

**FIG 3:**
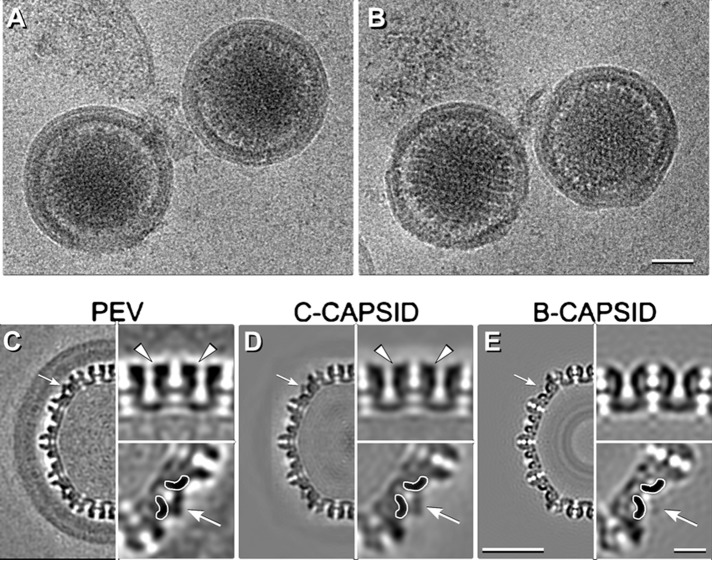
Cryo-EM reconstruction of PEVs. (A and B) Cryo-micrographs of intact PEVs. (C to E) Central sections of reconstructions of a PEV (C), a C-capsid (EMDB code 1354) (D), and an *in vitro*-matured B-capsid (27) (E). White arrows in panels C to E point to CCSC sites that are occupied in panels C and D but not in panel E. Symmetry-related regions are shown in magnified insets at bottom right in panels C to E. The triplexes to which the CCSC binds are delineated with white contours. The outcrops of density on hexamers indicative of pUL35 are marked with white arrowheads in panels C and D. Scale bars: panels B and D, 50 nm; inset in panel E, 10 nm.

We also imaged PEVs by cryo-EM, anticipating that three-dimensional (3D) reconstructions should give higher resolution for features conforming to icosahedral symmetry. Some intact PEVs are shown in Fig. 3A and B. In this data set, 93% of the PEVs contained C-capsids, with the remainder containing an A-capsid or a B-capsid. We calculated a 3D reconstruction by a projection-matching approach (Fig. 3C). The modest number of PEVs with intact envelopes and NEC layers limited the resolution to ~2 nm for the region containing the capsid and to ~3.5 nm for the whole particle (Fig. S5).

10.1128/mBio.00825-17.5 FIG S5  Resolution estimates for the PEV reconstruction. (A) Plot of the Fourier shell correlation coefficient. A mask that included only the region around the capsid shell was applied. (B) Resolution analysis of the PEV reconstruction within a 10-px (~5.7-nm)-thick shell. The resolution of the best regions is close to 2 nm. (C) Complete central section of the reconstruction in gray-scale. The outer layers of DNA, 2.5 nm apart, are resolved. (D) Local variations in resolution are distinguished by color coding. Download FIG S5, TIF file, 2 MB. Copyright © 2017 Newcomb et al. 2017 Newcomb et al. This content is distributed under the terms of the Creative Commons Attribution 4.0 International license.

We were particularly interested in whether PEVs contain pUL35 (VP26) and the CCSC, both of which are exposed on the capsid surface and were therefore *a priori* candidates for NEC binding sites. pUL35 binds after structural maturation of the procapsid creates its binding sites around the tips of the hexons, and it is present at full occupancy on A-capsids, B-capsids, and C-capsids (1). We found that it was also present at full occupancy on PEVs (cf. Fig. 3C and D). The CCSC did not bind to procapsids and was present at 10% to 15% occupancy on A-capsids and B-capsids and at ~60% occupancy on isolated C-capsids (6). Our reconstruction shows that it was present at full (~100%) occupancy on PEVs (Fig. 3C). Comparing this with the lower average occupancy on C-capsids (Fig. 3D) suggests that it is the C-capsids with the highest CCSC content that are taken up into PEVs and/or that additional binding of CCSC takes place during budding.

In the present context, care had to be taken to avoid imprinting features of the starting model—e.g., densities corresponding to the CCSC and pUL35—on the final reconstruction. To guard against this, our starting model was a reconstruction of *in vitro*-matured B-capsids, which are known to lack both the CCSC and pUL35 (27) (see Fig. 3E). Notwithstanding, both components showed up strongly in the reconstruction (Fig. 3C), which thus validated them as components of the PEV.

The NEC layer is conveyed as a shell ~8.5 nm thick, i.e., about twice as thick as the overlying envelope (Fig. 3C). It shows no sign of in-plane periodicity, affording further evidence that the ordered patches are not in register with the icosahedral lattice of the capsid and consequently were smeared laterally, giving a uniform sheet of density.

**FIG 4:**
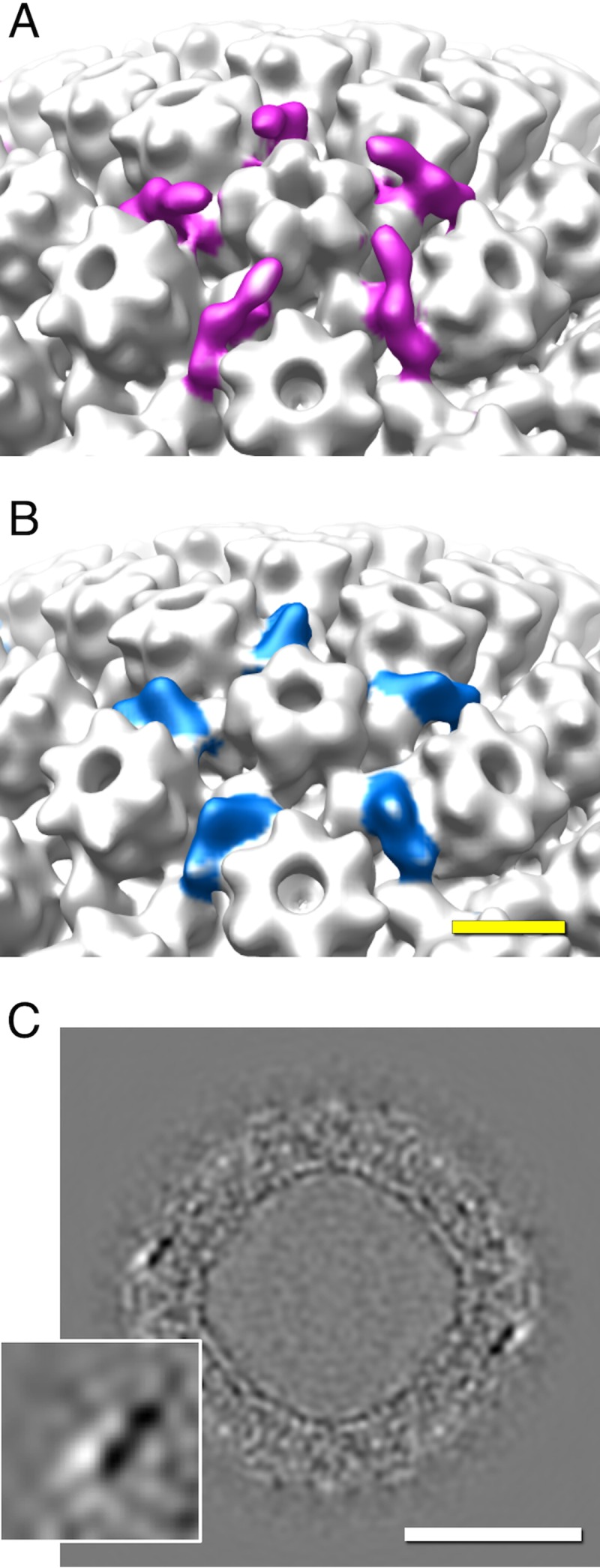
Structural changes in the CCSC upon being incorporated into a PEV. (A and B) Surface renderings of the surroundings of a vertex-located pUL19 penton with the CCSCs on a C-capsid (magenta in panel A) and a PEV (blue in panel B). (C) Grayscale section from the difference map, illustrating the change undergone by the CCSC (shown in the magnified inset at bottom left). Black, positive difference density; white, negative difference density. Scale bar = 50 nm.

The CCSC is a heterodimer of pUL17 and pUL25 (6). Of these, pUL17 is associated with a triplex and pUL25 extends toward the adjacent penton (7). The CCSC conformation in the PEV (Fig. 4B) differs from that in the C-capsid (Fig. 4A) in that part of pUL25 appears to be missing. This change is further illustrated in a difference density map in Fig. 4C. It represents a shift in the pUL25 location and/or an altered conformation, resulting from interactions between the NEC layer and the capsid as budding progresses.

### Connections of the NEC layer to the capsid.

**FIG 5:**
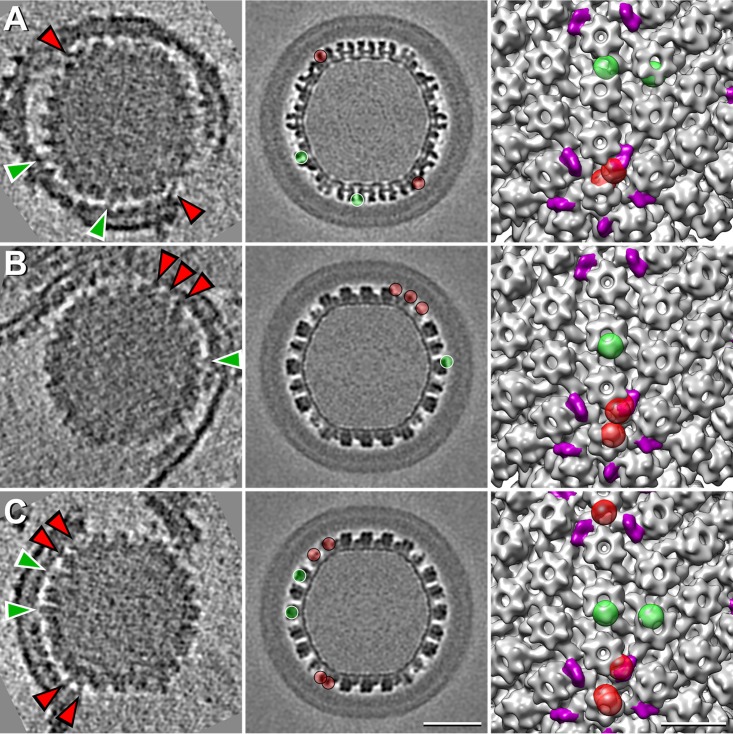
Densities bridging between the NEC layer and the capsid. (Left panels) Central tomographic slices from three virions. Some bridging densities are designated with arrowheads, red in the case of CCSC-associated densities and green for densities at non-CCSC sites. (Middle panels) Corresponding slices from the PEV cryo-EM reconstruction, with the sites of bridging densities marked with balls in same color coding as in the left-hand panel. (Right panels) Rendering of part of the C-capsid surface, with CCSC densities in magenta and sites of bridging densities in the tomograms (left-hand column) in the same color coding as in the middle panels. Scale bars = 50 nm (left and middle columns) and 20 nm (right column).

The NEC layer is anchored in the inner nuclear membrane by pUL34 molecules. PEV budding also involves interactions between the NEC layer and the capsid. Earlier studies proposed that the principal contacts are between pUL31 and the CCSC (15, 21). Because the NEC array is not in register with the capsid lattice, its features are smeared laterally in reconstructions based on the icosahedral symmetry of the capsid (see above). Accordingly, we decided to examine individual bridging densities visualized in tomograms (Fig. 5). As the bridging densities are small and the noise level is considerable, their identification was somewhat subjective. Accordingly, it was performed by three observers independently, and further analysis was restricted to sites selected by at least two observers.

Contact points on the capsid surface can be mapped by determining the orientation of a given PEV capsid and then rotating a reference C-capsid map into the same orientation and locating the corresponding slice and, hence, the point of contact. In this way, we were able to pick out some well-defined NEC-CCSC contacts (Fig. 5, red arrowheads in the left column and red balls in the middle and right-hand columns). Such contacts accounted for ~47% of the bridging densities picked, although they occupied only 25% of the capsid surface; i.e., their distribution was ~3-fold denser than at CCSC-remote sites. Some examples of the latter densities are indexed with green arrowheads and green balls in Fig. 5. The observers counted ~5 CCSC-contacting sites and ~5 other sites per PEV. In particular, there is no evidence of systematic contacts of NECs with pUL35, which decorates the outer rim of each hexon (white arrowheads in Fig. 3C and D). This observation is consistent with the fact that most A-capsids and B-capsids have full complements of pUL35 but remain in the nucleus.

### PEVs and membrane sacs.

In US3-null infections, the inner and outer membranes separate progressively, i.e., the PNS expands as PEVs accumulate, with the INM bulging into the nucleus (23, 24, 28). This creates an artificial compartment called a sac (see the introduction). The portion of a sac deriving from the inner nuclear membrane is rich in NECs (10).

**FIG 6:**
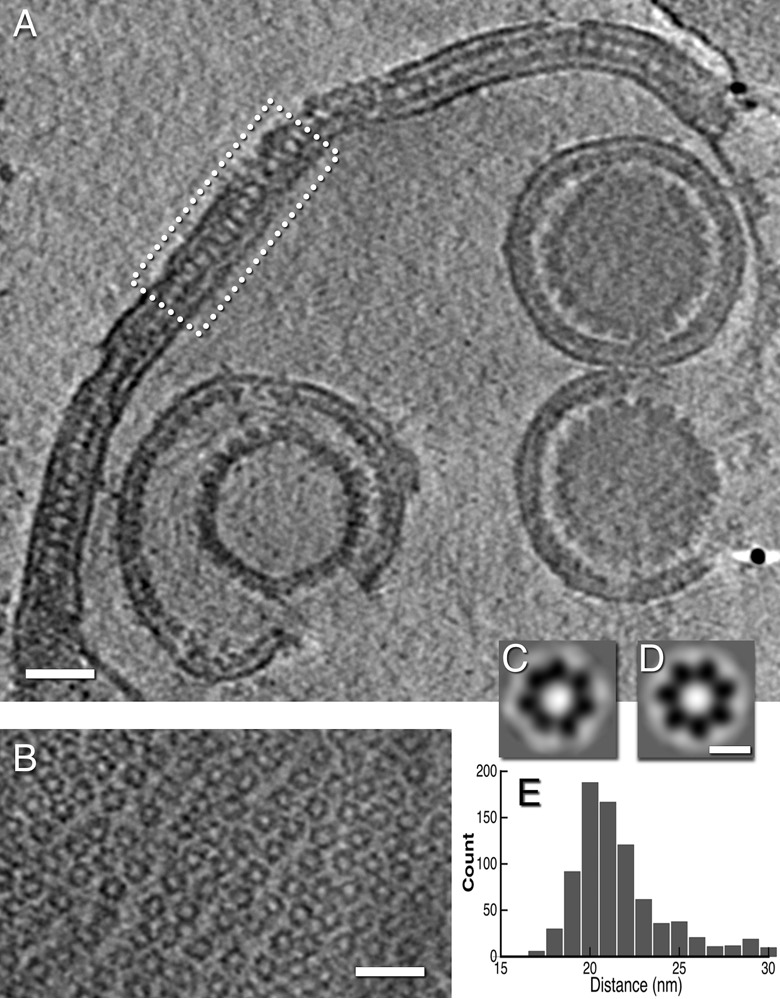
Arrays of NEC oligomers in a nuclear sac from a US3-null infection. (A) Slice through a cryo-electron tomogram of a sac. Part of the double lamella enclosing the sac is boxed. (B) A slice through part of the sac near the top of the tomogram showing a loosely ordered hexagonal array. (C and D) Averaged images of NEC rings, obtained without and with C7 symmetrization (see Materials and Methods). (E) Distribution of center-to-center spacings between neighboring rings. Scale bars = 50 nm (A and B) and 10 nm (C and D).

Numerous sacs or fragments thereof were present in our isolates. Some are bounded by a single membrane presumably derived from the ONM (data not shown), whereas others, such as the one shown in Fig. 6A, consisted of two lamellae paired back to back, with each lamella consisting of an INM plus a NEC layer. This sac encloses three PEVs, two of which contain C-capsids and the third an A-capsid. A tangential slice of the same tomogram (Fig. 6A) shows a quasihexagonal array of ring-like structures that we take to be NEC oligomers (Fig. 6B). The center-to-center spacings between these rings, at 17 to 22 nm (the distribution is shown in Fig. 6E), are larger and more variable than those seen with a PEV-associated honeycomb NEC array (~12 nm).

We used the ROTASTAT algorithms (29) to probe for rotational symmetries in the rings. This procedure estimates the likelihood of a given harmonic (order of symmetry) being statistically significant for the data set as a whole and does so at each radial position. The relatively weak signals from individual rings are boosted by being combined as conditional probabilities. ROTASTAT found a strongly expressed 7-fold symmetry at radii of 7.2 and 8.4 nm, i.e., around the periphery of the rings (Table 1). The only other symmetry to be detected was 6-fold at radii of 9.5 and 10.6 nm, where it refers to the surrounding six rings in the array. We conclude that the NEC rings in sacs have 7-fold symmetry. Averaged images of the heptamer ring are shown in Fig. 6C and D.

**TABLE 1:** Symmetry determination of NEC rings^a^

Radius (nm)	Orders significant by *t* test	Order (SRP value)
5.04		20 (5.60E–11)
		2 (3.26E–11)
		32 (8.93E–16)
6.16		36 (7.29E–20)
		34 (6.55E–21)
		2 (7.91E–22)
7.28	**7**	**7** (**1.02E+12.00**)
		4 (2.41E–19)
		2 (1.05E–25)
8.40	**7**	**7** (**3.06E+12.00**)
		4 (4.34E–14)
		6 (1.09E–21)
9.52	6	6 (25.03)
		7 (5.64E–8)
		4 (2.51E–17)
10.64	6	6 (6.25E+16.00)
		4 (7.85E–25)
		28 (1.00E–25)

a In ROTASTAT (29), significant orders of rotational symmetry are detected in two ways. In one approach, the spectral ratio product (SRP) is calculated whereby the ratio of the amplitude of a given harmonic at a given radius and the amplitude of the same harmonic for a similarly normalized piece of background are calculated for each ring in the data set (*n* = 188 in this case). These ratios are combined multiplicatively to give the SPR. If the ratios are, on average, even slightly less than unity, the SPR decays rapidly to very low values. With valid symmetries, the SPR remains above unity and may diverge to high values. The other approach is based on the Student’s *t* test. All orders of symmetry deemed statistically significant at a specified level (we used the conservative cutoff of 10^−6^) are output. The two approaches gave consistent results. Significant orders of symmetry in-ring are bolded. Number of particles, 188; number of backgrounds, 188; successive rings are at radii 2 px apart. Significance level = 1.0E^−6^. Orders with the three highest SRP values are given.

## DISCUSSION

**FIG 7:**
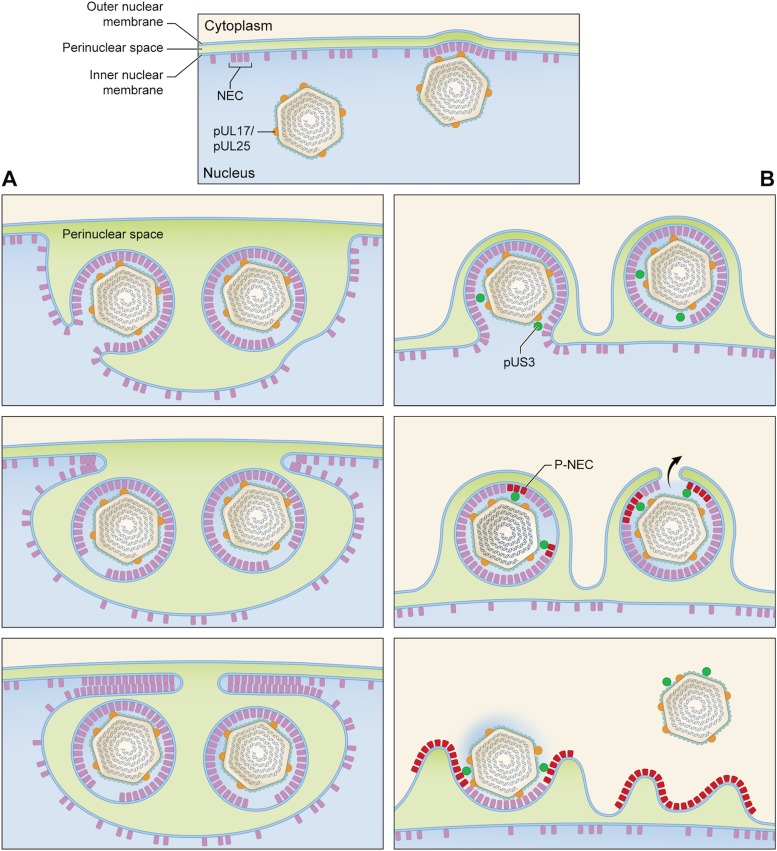
Pathways for nuclear egress in UL3-null and wild-type infections. In both cases, C-capsids start to bud at NEC-rich regions of the INM (top panel). (A) US3-null pathway leading to the formation of a nuclear sac. (B) Wild-type pathway. The top image in panel A shows one particle budded off with a “fontanelle” gap in its NEC layer and one particle at an advanced stage of budding. In the middle image, both PEVs have budded off and face-to-face interactions between apposing NEC layers are beginning to close off the sac. In the bottom image, this process has proceeded almost to completion. (B) In the wild-type pathway, we envisage that proteins entering the interstitial space between the NEC layer and capsid include the pUS3 kinase (green balls) and assume that there is no major structural difference between these PEVs and US3-null PEVs. Some pUS3 may be accommodated in the fontanelle gap; in any case, pUS3 should be sufficiently mobile to be able to access and phosphorylate the NEC layer (magenta-to-red switch), which would consequently disengage from the capsid. The fusion event that releases the capsid into the cytoplasm (middle image) would be facilitated by having the fontanelle membrane, which is presumably more pliable than the NEC-backed membrane, apposed to the ONM (black arrow). Finally (bottom image), the NEC proteins (P-NEC) are no longer bound to the capsid, which goes on to later stages of maturation, including acquisition of tegument proteins and secondary envelopment (not shown).

The HSV-1 egress pathway starts with exposure of patches of INM by phosphorylation of nuclear lamina proteins (30). This allows the NEC to bind to the INM. These NEC complexes recruit a capsid and pack together as the nascent PEV buds into the PNS. Finally, the PEV envelope fuses with the ONM, releasing the nucleocapsid into the cytoplasm. The scheme given in Fig. 7B draws on and is intended to update earlier accounts (31, 32).

In this study, we took advantage of the property that a mutation in the viral kinase US3 causes PEVs to accumulate in the perinuclear space, allowing them to be isolated in sufficient quantity for characterization by cryo-EM and cryo-ET. However, while thin-section electron micrographs of PEVs in cells infected with a US3-null PrV mutant showed them to be morphologically similar to the rarer PEVs observed in a wild-type infection (33) and to PEVs seen in gB/gH null infections (34), it remains possible that wild-type PEVs differ from US3-null PEVs in certain respects, including molecular composition. With this caveat, we conclude the following.

### Perturbation of the nuclear egress pathway.

On occasion, the pathway yields an aberrant assembly product that has no chance of maturing into an infectious extracellular virion. Such particles represent a fruitless biosynthetic investment. For example, although the PEV should contain a C-capsid, a significant minority have an A-capsid or a B-capsid (Fig. 2), thus lacking a genome. A mechanism that reduces such malfunctions is based on the higher CCSC content of C-capsids over that of A-capsids or B-capsids, which favors the selection of C-capsids for egress.

### PEVs and mature virions.

We find that PEVs differ from mature virions in almost every feature that is external to the capsid. Besides being ~20% larger, the mature virion envelope is densely studded with viral glycoproteins, whereas PEV envelopes have very few protruding densities (Fig. 1 and 2; see also Fig. S3 in the supplemental material). Such densities may, in principle, represent viral glycoproteins or cellular proteins. Glycoproteins gB and gH/gL mediate the fusion reaction whereby the mature virion enters a host cell and have been viewed as candidates for effectuating the fusion event that delivers the nucleocapsid into the cytoplasm. In the HSV-1 system, gB and gH have been reported to be separately dispensable without affecting nuclear egress, while deletion of both resulted in PEVs remaining in the PNS (34). In contrast, for PrV, deletion of both gB and gH had no effect on nuclear egress (35).

The gB spike has a distinctive morphology in its postfusion state, with three globular domains stacked radially (36), and is readily identifiable in tomograms (37). We saw only one PEV-associated spike from a total of 316 that was a plausible candidate for having that morphology, but we cannot exclude the possibility that PEVs have more gB in a different conformation. For example, ~12% of the PEV spikes could be compatible with prefusion gB (37) (J. Fontana, D. Atanasiu, W.T. Saw, J.R. Gallagher, R.G. Cox, J.C. Whitbeck, L.M. Brown, R.J. Eisenberg, G.H. Cohen, unpublished results), which presents in tomograms as shorter and less structurally differentiated than the postfusion molecule, making it difficult to identify with confidence. In future work, we plan to use immuno-EM to investigate the PEV contents of gB and other viral glycoproteins.

The tegument of a mature virion consists of at least 20 proteins in various amounts, irregularly distributed (38). In contrast, the PEV has a spherical shell of uniform thickness, composed of the NECs clustered in a honeycomb array interrupted only by the fontanelle gap (Fig. S4). Current data allow us to calculate that there are 2,500 to 3,000 copies of pUL31/pUL34 per PEV (see Text S1 in the supplemental material). Tomograms of mature virions show no sign of a NEC shell (Fig. 1C and D), and immunogold EM experiments did not detect either NEC protein (23), whence it follows that they disengage from the nucleocapsid as it enters the cytoplasm (see Fig. 7B).

10.1128/mBio.00825-17.6 TEXT S1  Copy numbers of pUL1 and pUL34. We estimate the complement of NEC heterodimers per PEV from the equation *N* ≅ *f* 24π*r*^2^/(√3/2*l*^2^), where *r* is the radius measured to the middle of the NEC layer (72.5 nm), *l* is the hexagonal lattice constant (12 nm), and *f* represents an incompleteness factor corresponding to the NEC layer, relating to the fontanelle and regions between neighboring patches. Taking a value of 0.9 to be a reasonable estimate for *f* gives a value for *N* of ~2,840 or, in round numbers, 2,600 to 3,000 for an array of hexamers and proportionally more for heptamers. Download TEXT S1, DOCX file, 0.05 MB. Copyright © 2017 Newcomb et al. 2017 Newcomb et al. This content is distributed under the terms of the Creative Commons Attribution 4.0 International license.

As for whether tegument proteins are already present in the PEV, the tomograms (e.g., Fig. 1E and F) and the cryo-EM (Fig. 3) consistently show that, if the NEC shell is discounted, there is little space to accommodate macromolecules in the region between capsid and envelope. A small amount of protein is present as bridging densities or in the fontanelle cavity (Fig. S4), but it did not exceed ~10% of the level that is present in a typical tegument (Text S2). Thus, while it appears that a few inner tegument proteins are acquired in the nucleus (reviewed in references 31 and 32), our results imply that the majority of tegument protein is acquired after the egressing nucleocapsid enters the cytoplasm.

10.1128/mBio.00825-17.7 TEXT S2  Volumes occupied by the tegument of a mature virion and by the corresponding compartment of a PEV. We approximate the volume occupied by tegument in a mature virion as the volume of a sphere of radius equal to the inner side of the membrane (94 nm) minus the volume of the capsid, approximated as a sphere of radius 61 nm. Similarly, we take the volume available for non-NEC components in a PEV as the volume of a sphere of a radius equal to that of the inner surface of the NEC layer (67 nm) minus the volume of the capsid. This varies with the size of a given mature virion (PEVs are more uniform in size) and gave a ratio of ~9:1. If the average protein densities in the two compartments are taken to be approximately equal, that implies that almost all of the tegument protein is acquired after the capsid leaves the nucleus. Download TEXT S2, DOCX file, 0.1 MB. Copyright © 2017 Newcomb et al. 2017 Newcomb et al. This content is distributed under the terms of the Creative Commons Attribution 4.0 International license.

Tegument proteins viewed as potential PEV components (31) include the pUS3 kinase and the abundant pUL48 (VP16) and pUL49 (VP22), each with ~700 copies (39). The case is persuasive for pUS3, which phosphorylates the NEC proteins (40) and—to access them—should be inside the PEV envelope. Moreover, pUS3 is present in mature virions (33). As for pUL49, immunogold EM experiments detected essentially no labeling of PEVs (41) but did observe some labeling for pUL48 (0.82 gold particles per PEV compared with 1.56 gold particles per mature virion). On the other hand, the pUL48 of PrV has been reported to be absent from PEVs (42).

### The NEC layer and its interaction with capsid.

Previous studies have shown that the NEC proteins can assemble into a honeycomb array with a center-to-center spacing of ~12 nm. Such a lattice was detected by cryo-ET of cells overexpressing the NEC proteins (10) and by 2D cryo-EM of NEC proteins bound *in vitro* to giant unilamellar vesicles (9) and has been modeled based on the crystal structure of the NEC heterodimer (12). Our negative-staining EM (Fig. 1) and cryo-EM (Fig. 3) data afford the first direct evidence for such a lattice in the context of a PEV. The data suggest that the NEC proteins form a spherical shell made up of hexagonal patches, interrupted by the fontanelle (Fig. S4). A similar gap in a spherical shell is left when the Gag layer of a budding HIV virion pinches off from its membrane (43).

These NEC arrays are packed with local hexagonal symmetry; to achieve closure, they should have faults of some kind. There is no indication that the NEC layer conforms to icosahedral symmetry, like the capsid, where 12 5-fold vertices allow closure (44). Rather, closure of the NEC shell appears to be achieved by having loosely coordinated patches with some voids at the interfaces between them.

We observed a 3-fold-higher incidence of “bridging densities” that involve interaction of the NEC layer with the CCSC (see also reference 21) than elsewhere on the capsid surface. Densities of both kinds (CCSC-proximal and non-proximal) averaged ~5 per PEV, although the totals may be somewhat higher, as our analysis was restricted to only the most clearly defined densities. On the other hand, some densities may not represent adhesive interactions but rather, the trapping of viral and/or cellular proteins between the NEC layer and the capsid as budding proceeds.

The NEC layer is a dominant feature of the PEV, but NEC proteins are not present in the mature virion (Fig. 1). It follows that they switch from a capsid-binding conformation to a nonbinding conformation during maturation. As cytoplasmic C-capsids have no NEC layer (45), we conclude that the NEC proteins dissociate from the capsid before or during its passage into the cytoplasm (Fig. 7B). It appears likely that this transition is induced by phosphorylation of the NEC complex by pUS3 (14, 46).

### Nuclear sacs.

There is now persuasive evidence that the egress pathway proceeds with the formation of an expanded region of PNS containing the PEV, followed by fusion of the PEV envelope with the ONM (Fig. 7B). However, in the absence of pUS3 as well as under some other circumstances in which egress from the PNS into the cytoplasm is impeded (24, 34), sacs are produced that bleb back from the nuclear envelope into the nucleus (Fig. 7A). It remains unclear how PEV assembly may be affected by this artificial remodeling of the perinuclear region of the cell. Nevertheless, sacs were recovered in our isolates from US3-null infections and most of them contained PEVs (e.g., Fig. 6A). Some of these sacs are bounded by a double lamella, each one consisting of a NEC layer overlying an INM-derived membrane. The lamellae are paired via back-to-back interactions between the two NEC layers. Earlier studies by thin-section EM, notably of US3-null infections, detected two-layered lamellar structures (see, e.g., references 22, 23, and 24). On the basis of the present data, we suggest that they represent paired NEC-lined membranes derived from the INM.

It appears that this NEC-NEC interaction drives remodeling of the nuclear envelope to create nuclear sacs. It is not yet clear whether PEVs contained within a sac can reenter the egress pathway and develop into infectious virions.

The tomograms reveal loosely ordered hexagonal arrays of ring-like oligomers that we take to be NEC proteins (Fig. 6B). The center-to-center spacing between rings is larger than in NEC arrays described earlier (9–12) and here (Fig. 1A and B). Hitherto, NEC arrays have been assumed to consist of hexamers. However, we find that the rings in sac lamellae are heptamers; as such, the arrays are not p6 lattices but random close packings (Fig. S2B). While the possibility that the same protein (NEC) might form different oligomers (hexamers, heptamers) under different conditions or in different locales has not been ruled out, the heptamer scenario has the following, possibly advantageous implication. If this symmetry were to occur in PEVs, there would be lower cooperativity to stabilize the array, easing its dispersal when the NEC layer separates from the nucleocapsid.

## MATERIALS AND METHODS

### Isolation of PEVs and sacs.

Typically, a 75-cm^2^ flask of Vero cells was infected at a multiplicity of infection (MOI) of 5 with a US3 deletion mutant, HSV-1(F)/us3(-) (a gift from Richard Roller, Department of Microbiology and Immunology, Carver College of Medicine, University of Iowa). At 12 h postinfection, overlay medium was decanted and the infected cell monolayer was washed with phosphate-buffered saline (PBS), scraped off, and pelleted. All PBS solutions used in this procedure contained protease inhibitor cocktail with no EDTA (Roche). The pellet was resuspended in 15 ml of cold hypotonic buffer (20 mM Tris, 20 mM KCl, 2 mM MgCl_2_, pH 7.5) where cells were allowed to swell for 20 min and then subjected to 10 strokes with a glass Dounce homogenizer. The resulting nuclei were pelleted at 1,800 rpm in a Sorvall Legend RT clinical centrifuge, resuspended in 1 ml of PBS, transferred to a 3-ml glass culture tube, disrupted by three 1-s pulses in a bath sonicator (Branson HD-50), and then centrifuged for 3 min at 6,000 rpm in a microcentrifuge (Eppendorf 5424). The resulting supernatant, designated supernatant 1, was removed and saved, while the pellet from this step was resuspended in 1 ml PBS, sonicated, and pelleted, leaving supernatant 2. This process was repeated to give supernatant 3. The supernatants were concentrated by pelleting and resuspension in 100 μl of PBS. In addition to nuclear debris, supernatant 1 contained primarily nucleocapsids plus a few PEVs; supernatant 2 contained fewer capsids but more PEVs and an occasional sac; and supernatant 3 contained fewer PEVs but more sacs than supernatant 2. The supernatant 2 concentrate was used to perform negative-staining EM, cryo-EM, and cryo-ET of PEVs and the supernatant 3 concentrate to perform cryo-ET of sacs.

### Western immunostaining.

Aliquots of supernatant 2 were layered onto 20% to 50% sucrose 10-step gradients made in Beckman Ultra-Clear tubes (5 by 41mm) and spun at 22,000 rpm in an SW 55Ti Beckman rotor for 45 min at 4°C. Fractions (1 drop each) were collected from the bottom of the centrifuge tube. Each fraction was diluted with PBS and examined by negative-staining EM to confirm the presence of PEVs. PEVs banded in the same location as mature virus, midway between the B-capsids and C-capsids seen in companion gradients (see Fig. S1 in the supplemental material). PEV-rich fractions were diluted with PBS and recentrifuged in 20% to 50% sucrose gradients as described above. Fractions were subjected to SDS-PAGE followed by Western blotting and probed (47) with pUL31-specific polyclonal antibody (Ul31-GST; a gift from Joel Baines, Louisiana State University [LSU] School of Veterinary Medicine) at a dilution of 1/2,000.

### Negative-staining EM and measurements of NEC layer spacings.

PEV-containing samples were stained with 1% uranyl acetate and examined in a CM120 microscope (FEI). Micrographs were recorded on a charge-coupled-device (CCD) camera (Gatan, Pleasanton, CA) at magnifications corresponding to pixel (px) sizes of 0.44, 0.33, and 0.26 nm. The repeat spacings in picket fence motifs seen in the NEC layer (see Results) were measured manually in real space by determining the length of a tract containing a certain number of repeats and dividing by that number. If a motif was long enough to form an arc with appreciable curvature, the data for two or three segments were summed.

### Cryo-electron tomography.

Isolates containing PEVs and sacs were mixed 2:1 with a suspension of 10-nm-diameter colloidal gold particles (Aurion, the Netherlands) to serve as fiducial markers, applied to glow-discharged EM grids (R 2/2 holey carbon grids; Quantifoil, Germany), and plunge-frozen using a Vitrobot (FEI, Hillsboro, OR). A Tecnai-12 microscope (FEI) operating at 120 keV and equipped with an energy filter (GIF 2002; Gatan, Warrendale, PA) set in zero-energy-loss mode with a slit width of 20 eV was used to record tilt series. Images were recorded on a CCD camera (Gatan, Warrendale, PA) (2,048 by 2,048 px) operated in semiautomated mode using SerialEM (48). The nominal defocus was ~4 µm, and a tilt series covered the range of −51° to +51° in 3° increments at a magnification of approximately ×53,500, giving a pixel size of 0.56 nm. The total electron dose for a tilt series was ~80 e/Å^2^, with a dose per projection of ~2.2 e/Å^2^. The Bsoft package (49) was used to calculate tomographic reconstructions and to extract virions as subtomograms. Subtomograms were denoised using the Gaussian Blur 3D filter implemented in ImageJ (50). In all, 35 cryo-tomograms were collected, containing a total of 101 PEVs. Forty-five percent of all PEVs were intact. A total of 9 sacs were imaged that contained 32 of our PEVs, most (75%) of which were intact.

As the bridging densities are small and the noise level is considerable, their identification is somewhat subjective. Accordingly, it was performed independently by three observers. Their pickings were distilled to sites selected by at least two observers. The CCSC-associated area above the capsid was defined as the region in the asymmetric unit demarcated by the penton and the 3 closest VP5 subunits of the adjacent P-hexon, representing approximately 25% of the capsid surface area. Of the connections picked by at least two observers, 47% fell within this area.

The averaged heptamers shown in Fig. 6C and D were obtained as follows. A total of 188 subtomograms, each containing a ring-like structure from the NEC array, were picked from an original tomogram. A tube with a circular cross-section of a size similar to that of the rings was generated for use as the initial reference. The subtomograms were aligned to this tube, and the orientations of the rings were refined through 10 iterations. The particles that correlated most strongly with the reference in each iteration were selected and averaged to calculate the values for next map. The final maps came from 99 particles.

### Cryo-electron microscopy.

Drops of sample (4 μl) were applied to EM grids bearing glow-discharged holey carbon films (see above), vitrified manually using a KF80 cryo-station (Reichert-Jung, Germany), and imaged with a CM200-FEG microscope (FEI) operated at 120 kV, as previously described (51). Images were recorded on a CCD camera (Gatan) (2,048 by 2,048 px) at a magnification of approximately ×25,000 (giving a pixel size of 5.7 Å) and processed with the Bsoft package (52), as previously described (53). Briefly, images of PEV were picked manually, yielding a total of 123 particles. These were contrast transfer function (CTF)-corrected by phase-flipping. Origins and orientations were determined by projection matching, using as a starting model a map of B-capsids obtained by *in vitro* maturation of purified procapsids (27). The final map was deposited in the EMDB (code 8607). Reconstructions were calculated using *breconstruct* (from the Bsoft package), an algorithm that integrates images as central sections of Fourier space and finally calculates an inverse Fourier transform. Using a cutoff criterion for forward scatter (FSC) of 0.3, the resolution of the reconstruction of C-capsid-containing PEVs was estimated at ~3.5 nm for the whole particle and at ~2 nm as calculated (54) for the region around the capsid shell. The resolution for a reconstruction from the few PEVs containing A-capsids or B-capsids (data not shown) was ~4.5 nm.

## References

[1] Cardone G, Heymann JB, Cheng N, Trus BL, Steven AC 2012 Procapsid assembly, maturation, nuclear exit: dynamic steps in the production of infectious herpesvirions. Adv Exp Med Biol 726:423–439. doi:10.1007/978-1-4614-0980-9_19. 22297525 PMC3475206

[2] Panté N, Kann M 2002 Nuclear pore complex is able to transport macromolecules with diameters of about 39 nm. Mol Biol Cell 13:425–434. doi:10.1091/mbc.01-06-0308. 11854401 PMC65638

[3] Skepper JN, Whiteley A, Browne H, Minson A 2001 Herpes simplex virus nucleocapsids mature to progeny virions by an envelopment → deenvelopment → reenvelopment pathway. J Virol 75:5697–5702. doi:10.1128/JVI.75.12.5697-5702.2001. 11356979 PMC114284

[4] Speese SD, Ashley J, Jokhi V, Nunnari J, Barria R, Li Y, Ataman B, Koon A, Chang YT, Li Q, Moore MJ, Budnik V 2012 Nuclear envelope budding enables large ribonucleoprotein particle export during synaptic Wnt signaling. Cell 149:832–846. doi:10.1016/j.cell.2012.03.032. 22579286 PMC3371233

[5] Rose A, Schlieker C 2012 Alternative nuclear transport for cellular protein quality control. Trends Cell Biol 22:509–514. doi:10.1016/j.tcb.2012.07.003. 22858153 PMC3462225

[6] Trus BL, Newcomb WW, Cheng N, Cardone G, Marekov L, Homa FL, Brown JC, Steven AC 2007 Allosteric signaling and a nuclear exit strategy: binding of UL25/UL17 heterodimers to DNA-filled HSV-1 capsids. Mol Cell 26:479–489. doi:10.1016/j.molcel.2007.04.010. 17531807 PMC1945812

[7] Toropova K, Huffman JB, Homa FL, Conway JF 2011 The herpes simplex virus 1 UL17 protein is the second constituent of the capsid vertex-specific component required for DNA packaging and retention. J Virol 85:7513–7522. doi:10.1128/JVI.00837-11. 21632758 PMC3147944

[8] Klupp BG, Granzow H, Keil GM, Mettenleiter TC 2006 The capsid-associated UL25 protein of the alphaherpesvirus pseudorabies virus is nonessential for cleavage and encapsidation of genomic DNA but is required for nuclear egress of capsids. J Virol 80:6235–6246. doi:10.1128/JVI.02662-05. 16775311 PMC1488961

[9] Bigalke JM, Heuser T, Nicastro D, Heldwein EE 2014 Membrane deformation and scission by the HSV-1 nuclear egress complex. Nat Commun 5:4131. doi:10.1038/ncomms5131. 24916797 PMC4105210

[10] Hagen C, Dent KC, Zeev-Ben-Mordehai T, Grange M, Bosse JB, Whittle C, Klupp BG, Siebert CA, Vasishtan D, Bäuerlein FJ, Cheleski J, Werner S, Guttmann P, Rehbein S, Henzler K, Demmerle J, Adler B, Koszinowski U, Schermelleh L, Schneider G, Enquist LW, Plitzko JM, Mettenleiter TC, Grünewald K 2015 Structural basis of vesicle formation at the inner nuclear membrane. Cell 163:1692–1701. doi:10.1016/j.cell.2015.11.029. 26687357 PMC4701712

[11] Bigalke JM, Heldwein EE 2015 Structural basis of membrane budding by the nuclear egress complex of herpesviruses. EMBO J 34:2921–2936. doi:10.15252/embj.201592359. 26511020 PMC4687684

[12] Zeev-Ben-Mordehai T, Weberruß M, Lorenz M, Cheleski J, Hellberg T, Whittle C, El Omari K, Vasishtan D, Dent KC, Harlos K, Franzke K, Hagen C, Klupp BG, Antonin W, Mettenleiter TC, Grünewald K 2015 Crystal structure of the herpesvirus nuclear egress complex provides insights into inner nuclear membrane remodeling. Cell Rep 13:2645–2652. doi:10.1016/j.celrep.2015.11.008. 26711332 PMC4700048

[13] Lye MF, Sharma M, El Omari K, Filman DJ, Schuermann JP, Hogle JM, Coen DM 2015 Unexpected features and mechanism of heterodimer formation of a herpesvirus nuclear egress complex. EMBO J 34:2937–2952. doi:10.15252/embj.201592651. 26511021 PMC4687685

[14] Yang K, Baines JD 2011 Selection of HSV capsids for envelopment involves interaction between capsid surface components pUL31, pUL17, and pUL25. Proc Natl Acad Sci U S A 108:14276–14281. doi:10.1073/pnas.1108564108. 21821792 PMC3161533

[15] Yang K, Wills E, Lim HY, Zhou ZH, Baines JD 2014 Association of herpes simplex virus pUL31 with capsid vertices and components of the capsid vertex-specific complex. J Virol 88:3815–3825. doi:10.1128/JVI.03175-13. 24453362 PMC3993549

[16] Leelawong M, Guo D, Smith GA 2011 A physical link between the pseudorabies virus capsid and the nuclear egress complex. J Virol 85:11675–11684. doi:10.1128/JVI.05614-11. 21880751 PMC3209322

[17] Ye GJ, Roizman B 2000 The essential protein encoded by the UL31 gene of herpes simplex virus 1 depends for its stability on the presence of UL34 protein. Proc Natl Acad Sci U S A 97:11002–11007. doi:10.1073/pnas.97.20.11002. 11005871 PMC27138

[18] Stackpole CW 1969 Herpes-type virus of the frog renal adenocarcinoma. I. Virus development in tumor transplants maintained at low temperature. J Virol 4:75–93. 5808113 10.1128/jvi.4.1.75-93.1969 PMC375840

[19] Granzow H, Klupp BG, Fuchs W, Veits J, Osterrieder N, Mettenleiter TC 2001 Egress of alphaherpesviruses: comparative ultrastructural study. J Virol 75:3675–3684. doi:10.1128/JVI.75.8.3675-3684.2001. 11264357 PMC114859

[20] Padula ME, Sydnor ML, Wilson DW 2009 Isolation and preliminary characterization of herpes simplex virus 1 primary enveloped virions from the perinuclear space. J Virol 83:4757–4765. doi:10.1128/JVI.01927-08. 19279117 PMC2682069

[21] Baines JD, Hsieh CE, Wills E, Mannella C, Marko M 2007 Electron tomography of nascent herpes simplex virus virions. J Virol 81:2726–2735. doi:10.1128/JVI.02571-06. 17215293 PMC1865967

[22] Klupp BG, Granzow H, Mettenleiter TC 2001 Effect of the pseudorabies virus US3 protein on nuclear membrane localization of the UL34 protein and virus egress from the nucleus. J Gen Virol 82:2363–2371. doi:10.1099/0022-1317-82-10-2363. 11562530

[23] Reynolds AE, Wills EG, Roller RJ, Ryckman BJ, Baines JD 2002 Ultrastructural localization of the herpes simplex virus type 1 UL31, UL34, and US3 proteins suggests specific roles in primary envelopment and egress of nucleocapsids. J Virol 76:8939–8952. doi:10.1128/JVI.76.17.8939-8952.2002. 12163613 PMC136992

[24] Ryckman BJ, Roller RJ 2004 Herpes simplex virus type 1 primary envelopment: UL34 protein modification and the US3-UL34 catalytic relationship. J Virol 78:399–412. doi:10.1128/JVI.78.1.399-412.2004. 14671121 PMC303423

[25] Liu Z, Kato A, Oyama M, Kozuka-Hata H, Arii J, Kawaguchi Y 2015 Role of host cell p32 in herpes simplex virus 1 de-envelopment during viral nuclear egress. J Virol 89:8982–8998. doi:10.1128/JVI.01220-15. 26085152 PMC4524097

[26] Grünewald K, Desai P, Winkler DC, Heymann JB, Belnap DM, Baumeister W, Steven AC 2003 Three-dimensional structure of herpes simplex virus from cryo-electron tomography. Science 302:1396–1398. doi:10.1126/science.1090284. 14631040

[27] Aksyuk AA, Newcomb WW, Cheng N, Winkler DC, Fontana J, Heymann JB, Steven AC 2015 Subassemblies and asymmetry in assembly of herpes simplex virus procapsid. mBio 6:e01525-15. doi:10.1128/mBio.01525-15. 26443463 PMC4611051

[28] Fuchs W, Klupp BG, Granzow H, Osterrieder N, Mettenleiter TC 2002 The interacting UL31 and UL34 gene products of pseudorabies virus are involved in egress from the host-cell nucleus and represent components of primary enveloped but not mature virions. J Virol 76:364–378. doi:10.1128/JVI.76.1.364-378.2002. 11739701 PMC135715

[29] Kocsis E, Cerritelli ME, Trus BL, Cheng N, Steven AC 1995 Improved methods for determination of rotational symmetries in macromolecules. Ultramicroscopy 60:219–228. doi:10.1016/0304-3991(95)00070-2. 7502382

[30] Leach NR, Roller RJ 2010 Significance of host cell kinases in herpes simplex virus type 1 egress and lamin-associated protein disassembly from the nuclear lamina. Virology 406:127–137. doi:10.1016/j.virol.2010.07.002. 20674954 PMC2948959

[31] Johnson DC, Baines JD 2011 Herpesviruses remodel host membranes for virus egress. Nat Rev Microbiol 9:382–394. doi:10.1038/nrmicro2559. 21494278

[32] Hellberg T, Paßvogel L, Schulz KS, Klupp BG, Mettenleiter TC 2016 Nuclear egress of herpesviruses: the prototypic vesicular nucleocytoplasmic transport. Adv Virus Res 94:81–140. doi:10.1016/bs.aivir.2015.10.002. 26997591

[33] Granzow H, Klupp BG, Mettenleiter TC 2004 The pseudorabies virus US3 protein is a component of primary and of mature virions. J Virol 78:1314–1323. doi:10.1128/JVI.78.3.1314-1323.2004. 14722286 PMC321416

[34] Farnsworth A, Wisner TW, Webb M, Roller R, Cohen G, Eisenberg R, Johnson DC 2007 Herpes simplex virus glycoproteins gB and gH function in fusion between the virion envelope and the outer nuclear membrane. Proc Natl Acad Sci U S A 104:10187–10192. doi:10.1073/pnas.0703790104. 17548810 PMC1891206

[35] Klupp B, Altenschmidt J, Granzow H, Fuchs W, Mettenleiter TC 2008 Glycoproteins required for entry are not necessary for egress of pseudorabies virus. J Virol 82:6299–6309. doi:10.1128/JVI.00386-08. 18417564 PMC2447092

[36] Heldwein EE, Lou H, Bender FC, Cohen GH, Eisenberg RJ, Harrison SC 2006 Crystal structure of glycoprotein B from herpes simplex virus 1. Science 313:217–220. doi:10.1126/science.1126548. 16840698

[37] Zeev-Ben-Mordehai T, Vasishtan D, Hernández Durán A, Vollmer B, White P, Prasad Pandurangan A, Siebert CA, Topf M, Grünewald K 2016 Two distinct trimeric conformations of natively membrane-anchored full-length herpes simplex virus 1 glycoprotein B. Proc Natl Acad Sci U S A 113:4176–4181. doi:10.1073/pnas.1523234113. 27035968 PMC4839410

[38] Newcomb WW, Brown JC 2010 Structure and capsid association of the herpesvirus large tegument protein UL36. J Virol 84:9408–9414. doi:10.1128/JVI.00361-10. 20631146 PMC2937621

[39] Newcomb WW, Jones LM, Dee A, Chaudhry F, Brown JC 2012 Role of a reducing environment in disassembly of the herpesvirus tegument. Virology 431:71–79. doi:10.1016/j.virol.2012.05.017. 22695308

[40] Wisner TW, Wright CC, Kato A, Kawaguchi Y, Mou F, Baines JD, Roller RJ, Johnson DC 2009 Herpesvirus gB-induced fusion between the virion envelope and outer nuclear membrane during virus egress is regulated by the viral US3 kinase. J Virol 83:3115–3126. doi:10.1128/JVI.01462-08. 19158241 PMC2655551

[41] Naldinho-Souto R, Browne H, Minson T 2006 Herpes simplex virus tegument protein VP16 is a component of primary enveloped virions. J Virol 80:2582–2584. doi:10.1128/JVI.80.5.2582-2584.2006. 16474165 PMC1395364

[42] Fuchs W, Granzow H, Klupp BG, Kopp M, Mettenleiter TC 2002 The UL48 tegument protein of pseudorabies virus is critical for intracytoplasmic assembly of infectious virions. J Virol 76:6729–6742. doi:10.1128/JVI.76.13.6729-6742.2002. 12050386 PMC136261

[43] Carlson LA, Hurley JH 2012 In vitro reconstitution of the ordered assembly of the endosomal sorting complex required for transport at membrane-bound HIV-1 gag clusters. Proc Natl Acad Sci U S A 109:16928–16933. doi:10.1073/pnas.1211759109. 23027949 PMC3479502

[44] Caspar DL, Klug A 1962 Physical principles in the construction of regular viruses. Cold Spring Harb Symp Quant Biol 27:1–24. doi:10.1101/SQB.1962.027.001.005. 14019094

[45] Fan WH, Roberts AP, McElwee M, Bhella D, Rixon FJ, Lauder R 2015 The large tegument protein pUL36 is essential for formation of the capsid vertex-specific component at the capsid-tegument interface of herpes simplex virus 1. J Virol 89:1502–1511. doi:10.1128/JVI.02887-14. 25410861 PMC4300765

[46] Mou F, Wills E, Baines JD 2009 Phosphorylation of the U_L_31 protein of herpes simplex virus 1 by the U_S_3-encoded kinase regulates localization of the nuclear envelopment complex and egress of nucleocapsids. J Virol 83:5181–5191. doi:10.1128/JVI.00090-09. 19279109 PMC2682108

[47] Newcomb WW, Booy FP, Brown JC 2007 Uncoating the herpes simplex virus genome. J Mol Biol 370:633–642. doi:10.1016/j.jmb.2007.05.023. 17540405 PMC1975772

[48] Mastronarde DN 2005 Automated electron microscope tomography using robust prediction of specimen movements. J Struct Biol 152:36–51. doi:10.1016/j.jsb.2005.07.007. 16182563

[49] Heymann JB, Cardone G, Winkler DC, Steven AC 2008 Computational resources for cryo-electron tomography in Bsoft. J Struct Biol 161:232–242. doi:10.1016/j.jsb.2007.08.002. 17869539 PMC2409064

[50] Schneider CA, Rasband WS, Eliceiri KW 2012 NIH Image to ImageJ: 25 years of image analysis. Nat Methods 9:671–675. doi:10.1038/nmeth.2089. 22930834 PMC5554542

[51] Cheng N, Conway JF, Watts NR, Hainfeld JF, Joshi V, Powell RD, Stahl SJ, Wingfield PE, Steven AC 1999 Tetrairidium, a four-atom cluster, is readily visible as a density label in three-dimensional cryo-EM maps of proteins at 10–25 Å resolution. J Struct Biol 127:169–176. doi:10.1006/jsbi.1999.4120. 10527906

[52] Heymann JB, Belnap DM 2007 Bsoft: image processing and molecular modeling for electron microscopy. J Struct Biol 157:3–18. doi:10.1016/j.jsb.2006.06.006. 17011211

[53] McHugh CA, Fontana J, Nemecek D, Cheng N, Aksyuk AA, Heymann JB, Winkler DC, Lam AS, Wall JS, Steven AC, Hoiczyk E 2014 A virus capsid-like nanocompartment that stores iron and protects bacteria from oxidative stress. EMBO J 33:1896–1911. doi:10.15252/embj.201488566. 25024436 PMC4195785

[54] Cardone G, Heymann JB, Steven AC 2013 One number does not fit all: mapping local variations in resolution in cryo-EM reconstructions. J Struct Biol 184:226–236. doi:10.1016/j.jsb.2013.08.002. 23954653 PMC3837392

