# Supplementary figures and images for: The Primary Enveloped Virion of Herpes Simplex Virus 1: Its Role in Nuclear Egress
Source: mBio. 2017 Jun 13;8(3):e00825-17. doi: 10.1128/mBio.00825-17 (PMC5472190; doi:10.1128/mBio.00825-17)

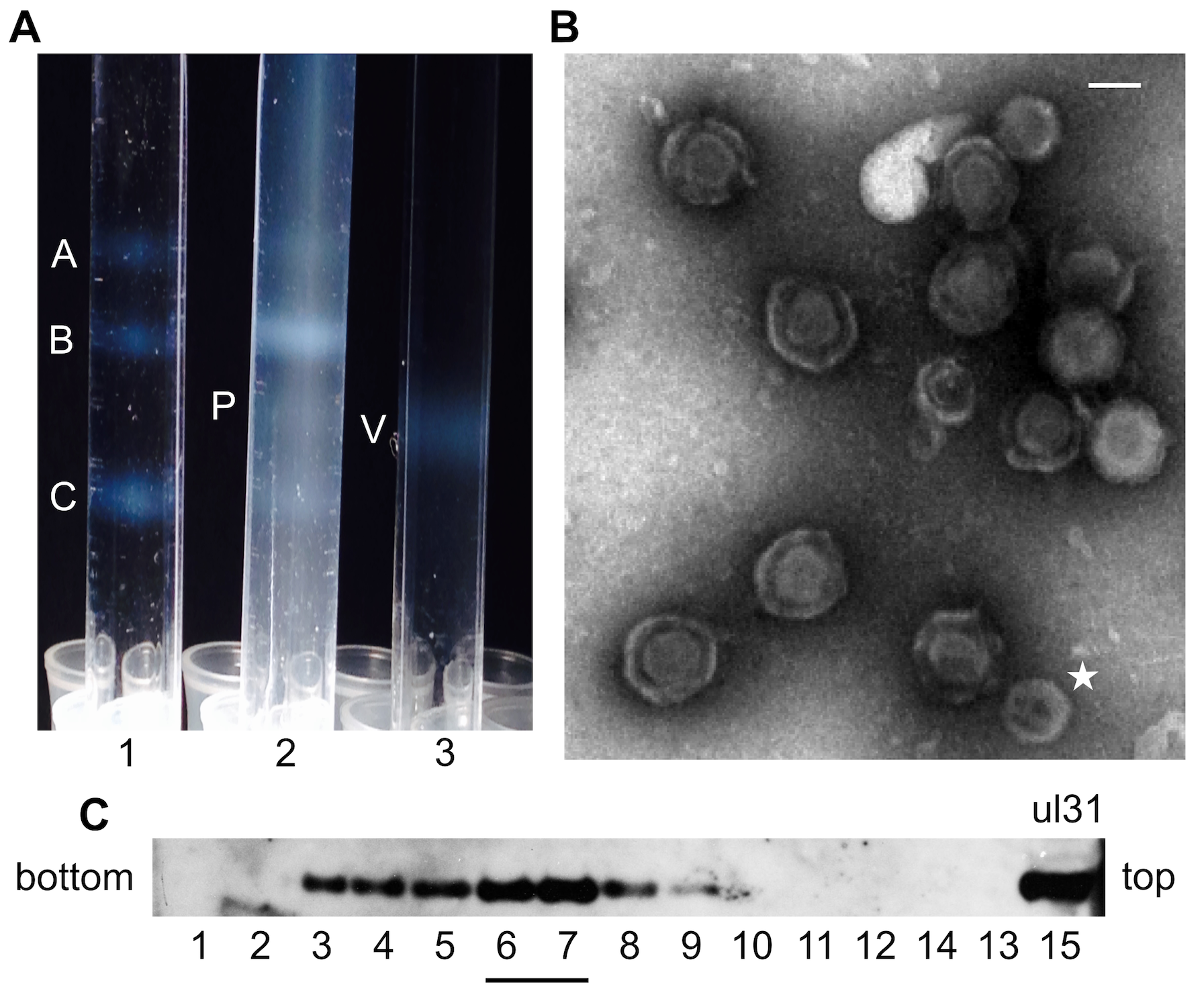

Supplement: FIG S1 [file mbo003173345sf1.tif]

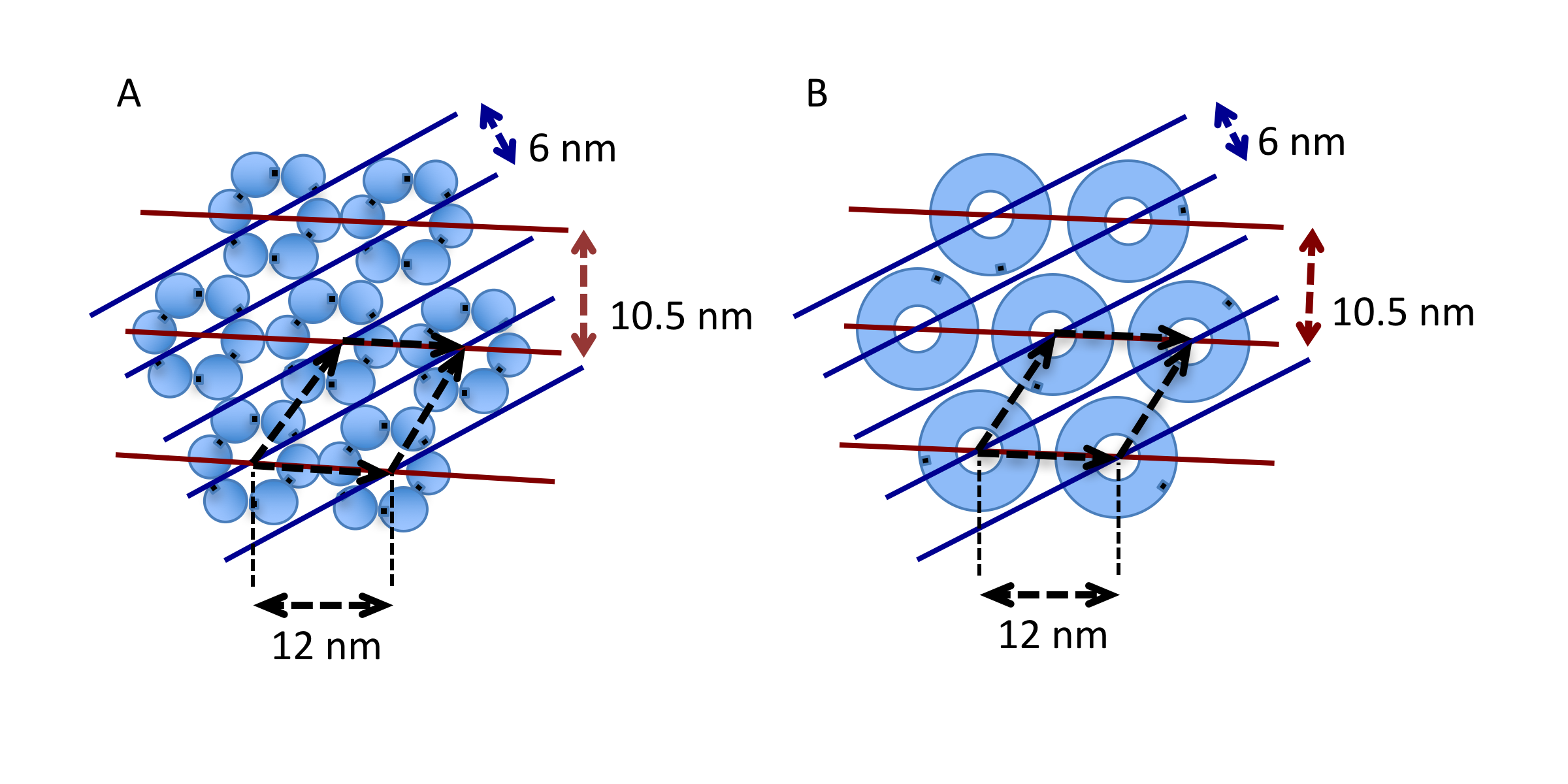

Supplement: FIG S2 [file mbo003173345sf2.tif]

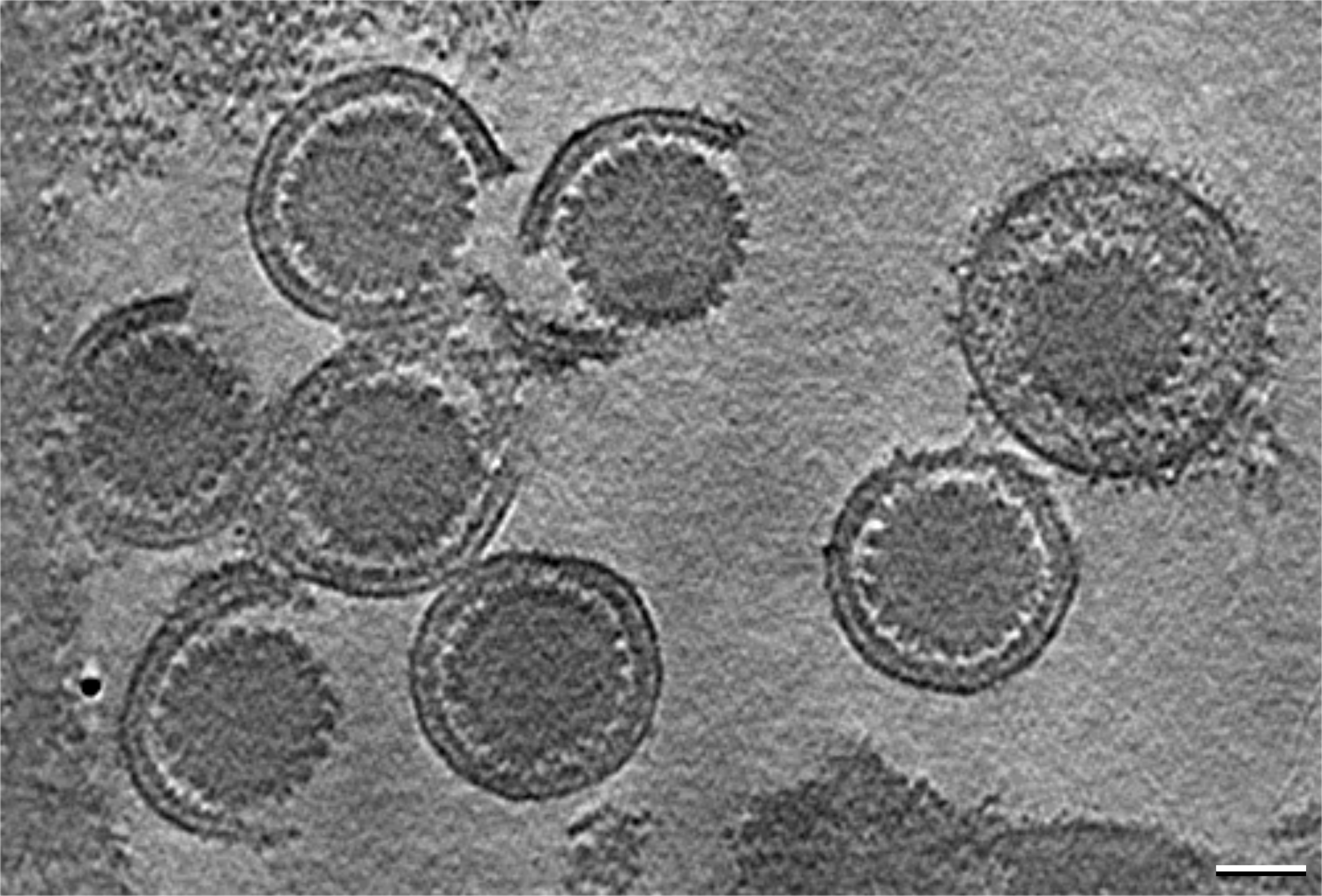

Supplement: FIG S3 [file mbo003173345sf3.tif]

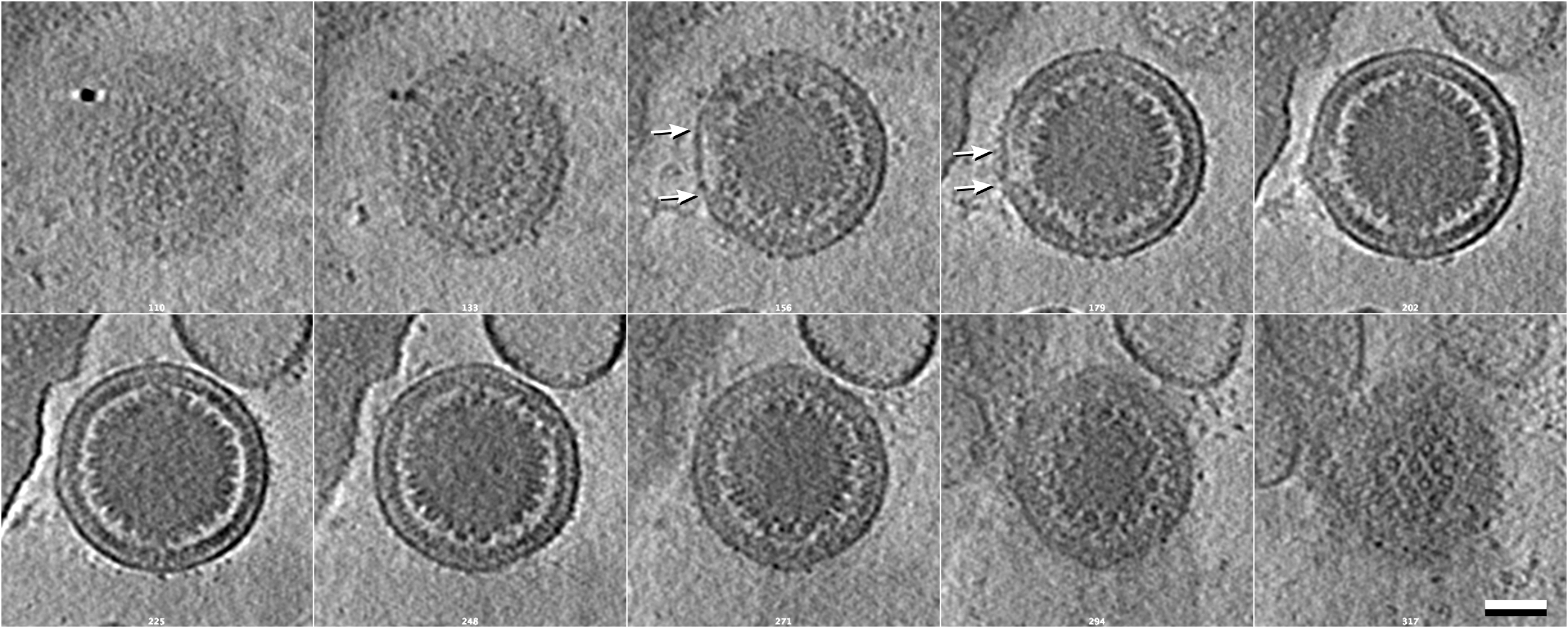

Supplement: FIG S4 [file mbo003173345sf4.tif]

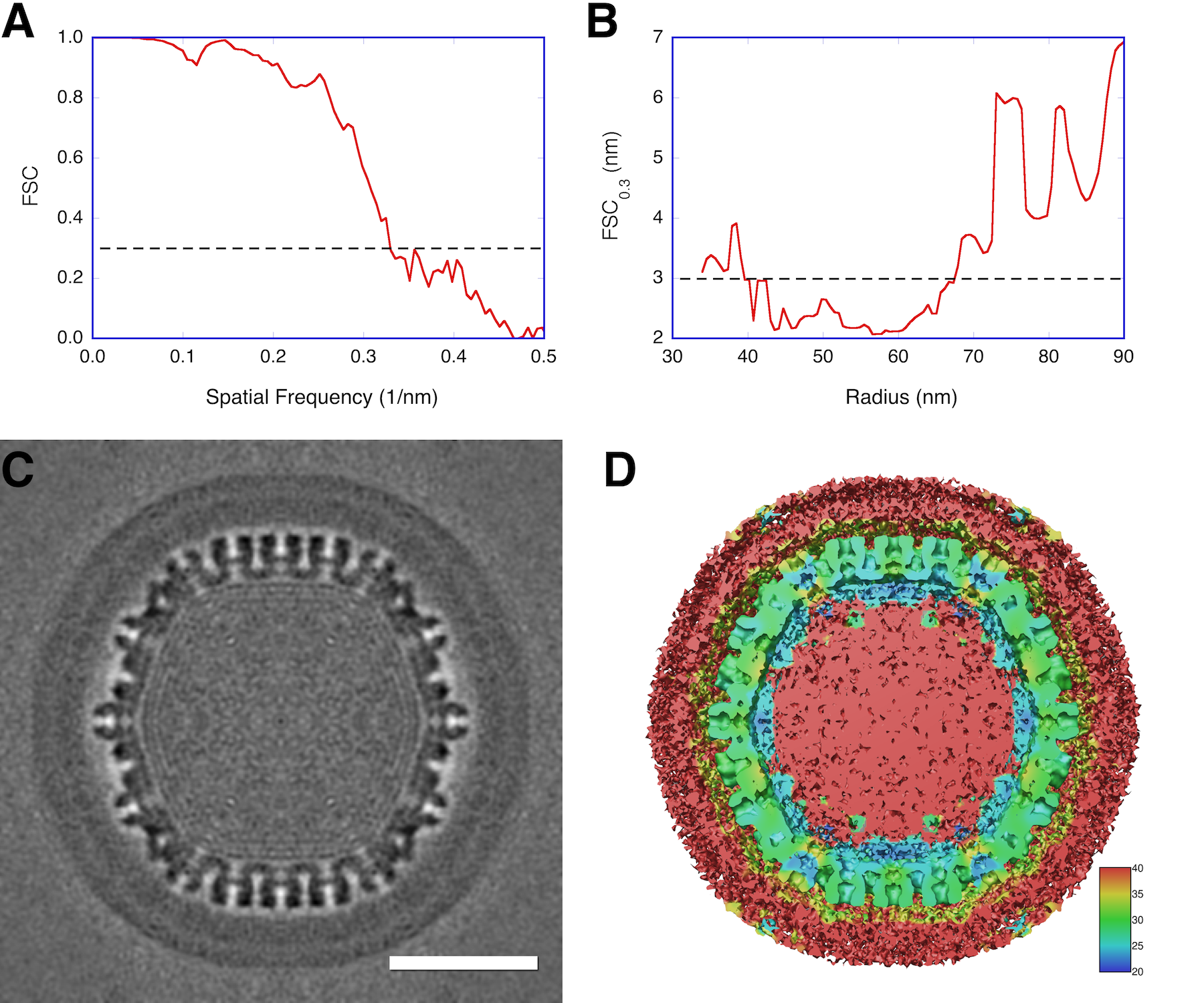

Supplement: FIG S5 [file mbo003173345sf5.tif]
