# Supplementary material for: The Primary Enveloped Virion of Herpes Simplex Virus 1: Its Role in Nuclear Egress
Source: mBio. 2017 Jun 13;8(3):e00825-17. doi: 10.1128/mBio.00825-17 (PMC5472190; doi:10.1128/mBio.00825-17)
Supplement: TEXT S1 [file mbo003173345s1.docx]

**Text S1. Copy numbers of pUL1 and pUL34.** We estimate the complement of NEC heterodimers per PEV from the equation: N ≅ f. 24π. r^2^/ (√3/2.*l*^2^) where r is the radius measured to the middle of the NEC layer (72.5 nm), *l* is the hexagonal lattice constant (12 nm), and f represents an incompleteness factor of the NEC layer, relating to the fontanelle and regions between neighboring patches. Taking a value of 0.9 to be a reasonable estimate for f gives a value for N of ~ 2840 or, in round numbers, 2600 – 3000 for an array of hexamers and proportionally more for heptamers.
