# Supplementary material for: The Primary Enveloped Virion of Herpes Simplex Virus 1: Its Role in Nuclear Egress
Source: mBio. 2017 Jun 13;8(3):e00825-17. doi: 10.1128/mBio.00825-17 (PMC5472190; doi:10.1128/mBio.00825-17)
Supplement: TEXT S2 [file mbo003173345s2.docx]

**Text S2. Volumes occupied by the tegument of a mature virion and by the corresponding compartment of a PEV.** We approximate the volume occupied by tegument in a mature virion as the volume of a sphere of radius equal to the inside of the membrane (94 nm) minus the volume of the capsid, approximated as a sphere of radius 61 nm. Similarly, we take the volume available for non-NEC components in a PEV as the volume of a sphere of radius equal to that of the inner surface of the NEC layer (67 nm) minus the volume of the capsid. This varies with the size of a given mature virion (PEVs are more uniform in size), and gave a ratio of ~ 9 : 1. If the average protein density in the two compartments is taken to be approximately equal, that implies that almost all of the tegument protein is acquired after the capsid leaves the nucleus.
